# Correspondence

**Published:** 1901-05

**Authors:** R. R. Kime

**Affiliations:** Chairman Tri-State Committee


### CORRESPONDENCE.

---

*Editor Journal–Record of Medicine:* The sociological committee of the Tri-State Medical Society desires the support of the medical profession of each of the three States which it represents, Alabama, Georgia and Tennessee.

The subject will be presented at next meeting of each State Medical Society by the following physicians:

Tennessee by W. G. Bogart, M.D.; Alabama by J. E. Purdon, M.D.; Georgia by J. S. Todd, M.D., T. O. Powell, M.D., Drs. Childs & Champion, R. R. Kime, M.D.

We hope to get a committee appointed from each State society to work in conjunction with the Tri-State Medical Society.

It is the desire of the committee to get unity of action in all three of the States. To this end we invite the cooperation of the profession.

R. R. KIME,
Chairman Tri-State Committee.

